# The Total Artificial Heart in End-Stage Congenital Heart Disease

**DOI:** 10.3389/fphys.2017.00131

**Published:** 2017-05-09

**Authors:** Chet R. Villa, David L. S. Morales

**Affiliations:** Cincinnati Children's Hospital Medical Center, Heart InstituteCincinnati, OH, USA

**Keywords:** total artificial heart, congenital heart disease, mechanical circulatory support, pediatrics, bridge to transplantation

## Abstract

The development of durable ventricular assist devices (VADs) has improved mortality rates and quality of life in patients with end stage heart failure. While the use of VADs has increased dramatically in recent years, there is limited experience with VAD implantation in patients with complex congenital heart disease (CHD), despite the fact that the number of patients with end stage CHD has grown due to improvements in surgical and medical care. VAD use has been limited in patients with CHD and end stage heart failure due to anatomic (systemic right ventricle, single ventricle, surgically altered anatomy, valve dysfunction, etc.) and physiologic constraints (diastolic dysfunction). The total artificial heart (TAH), which has right and left sided pumps that can be arranged in a variety of orientations, can accommodate the anatomic variation present in CHD patients. This review provides an overview of the potential use of the TAH in patients with CHD.

## Background

Ventricular assist devices (VADs) have been shown to improve mortality and quality of life in adults with refractory heart failure. This has led to a dramatic increase in VAD use among adults over the last decade (Kirklin et al., 2014). Recent reports have shown a dramatic increase in VAD use in children as well (Almond et al., 2013; Villa et al., 2017) as ~20% of pediatric patients are bridged to transplant with a VAD in the current era (Dipchand et al., 2014). These trends in VAD utilization have in turn driven improvements in waitlist mortality for both children (Zafar et al., 2015a) and adults (Emin et al., 2013). Despite the improvement in survival and quality of life brought about by improvements in VAD technology, patients with congenital heart disease (CHD) are less likely to receive a VAD while on the waitlist and are more likely to die while awaiting transplantation (Gelow et al., 2013; Zafar et al., 2015a). The need for improved support options for patients with CHD is significant as ~45% of pediatric heart transplant recipients have CHD (Dipchand et al., 2014) and the number of adults with CHD who experience heart failure (Khairy et al., 2010) or require transplant (Lund et al., 2014) is continuing to grow. Historically, VAD use has not been extended to patients with congenital disease due to multiple factors including patient size, anatomic complexity, multi-organ dysfunction (Kiesewetter et al., 2007; Dimopoulos et al., 2008; Ridderbos et al., 2015) and complex physiology including both systolic (Piran et al., 2002) and diastolic dysfunction (Gewillig et al., 1992). Data is starting to be collected regarding the use of VADs in patients with CHD, however, the current literature is limited to single center case series/case reports. This review will discuss the physiology underlying end stage heart disease with a focus on the potential use of the total artificial heart (TAH).

A few proprietary artificial heart devices have been developed including the Syncardia TAH (Syncardia, Tucson, AZ, USA), AbioCor (Abiomed, Danvers, Massachusetts, USA), and CARMAT (Carmat, Velizy, France). The history of these devices and their clinical use has recently been reviewed (Gerosa et al., 2014). Among the devices listed, the Syncardia (TAH) is the only device currently in use. The CARMAT is currently undergoing feasibility studies, however, data has been limited to a handful of patients thus far (Carpentier et al., 2015). There is also no data on the use of the AbioCor (Dowling et al., 2004) or CARMAT in patients with CHD. Given the paucity of data regarding the use of the AbioCor and CARMAT devices, this review will focus on the use of the Syncardia TAH.

The (TAH) was developed for use in adults with end-stage heart failure who had a contraindication to LVAD or biventricular VAD (BiVAD) (Copeland et al., 2004) including valve insufficiency, intractable arrhythmias and ventricular clot. Device use has subsequently been expanded to younger patients (Leprince et al., 2005; Ryan et al., 2015) and patients with CHD (Morales et al., 2012; Kirsch et al., 2013; Rossano et al., 2014; Ryan et al., 2015). The use of the TAH for complex CHD is potentially paradigm shifting for selected patients. Congenital heart patients with end-stage heart failure who have residual lesions (i.e., VSD, severe semilunar valve insufficiency, stenotic right ventricular to pulmonary artery conduit) that would have to be addressed in order to place a VAD or BiVAD clearly have a different mortality and morbidity profile when compared to patients with cardiomyopathy alone (Zafar et al., 2015b). It is in these patients that the TAH may simplify and optimize support rather than placing a VAD or BiVAD with concurrent cardiac procedures (i.e., VSD closure, conduit revision, aortic valve replacement). The current published experience with the TAH in CHD is listed in Table 1. The range of physiologies (single ventricle, two ventricle) and anatomic abnormalities represented shows the potential for the TAH to address multiple anatomic considerations with implantation alone. The TAH also has the ability to markedly improve patient symptoms and end organ dysfunction in anatomies/physiologies where medical management has shown limited or no benefit (i.e., diastolic dysfunction, CHD (Kouatli et al., 1997; Shaddy et al., 2007; van der Bom et al., 2013, etc.) and VAD options are limited (Table 2).

**Table 1:** **Clinical experience with the total artificial heart (TAH) in patients in congenital heart disease**.

**References**	**Anatomy**	**Duration of support**	**Surgical considerations with TAH implantation**	**Outcome**
Morales et al., 2012	Dextrocardia, {S,L,L} ccTGA status post Rastelli with severe aortic insufficiency and obstruction of left ventricle-pulmonary artery conduit	160 days	Separation of right and left pumps with parallel orientation	Bridged to transplantation
Rossano et al., 2014	Pulmonary atresia with intact ventricular septum status post Fontan	61 days	Creation of “neo-right atrium”	Bridged to transplantation
Si et al., 2015	Dextrocardia, {S,L,L} ccTGA, ventricular septal defect, pulmonary stenosis and Ebsteinoid tricuspid valve status post Senning-Rastelli with severe tricuspid regurgitation	8 days	Reversal of Senning	Bridged to transplantation

*ccTGA, congenitally corrected transposition of the great arteries*.

**Table 2:** **Potential indications for total artificial heart (TAH)**.

**Failing congenital heart disease**
Residual anatomic lesions with coexisting cardiac dysfunction
Fontan (Rossano et al., 2014)
Systemic right ventricular failure (Morales et al., 2012)
Transposition of the great arteries (TGA), status post Mustard or Senning
Congenitally corrected transposition of the great arteries (ccTGA)
**Alternate clinical scenarios** (Copeland et al., 2004)
Allograft failure
Biventricular heart failure
Cardiac tumor
Chronic right ventricular failure with existing left ventricular assist device
Intractable arrhythmias
Active malignancy receiving cardiotoxic therapies
Restrictive cardiomyopathy
Ventricular clot

## Congenital heart disease in the two ventricle circulation

Patients with a systemic right ventricle due to transposition of the great arteries (TGA) or congenitally corrected transposition of the great arteries (ccTGA) carry a life-long risk of systemic right ventricular dysfunction (Graham et al., 2000; Vejlstrup et al., 2015) and heart failure. The development of systemic ventricular dysfunction is reflected in the growing number of case series describing the outcome of VAD support for these patients (Joyce et al., 2010; Peng et al., 2014; Maly et al., 2015). While most centers have described morphologic right (systemic) ventricular support alone, the majority of patients will have coincident morphologic left ventricular (sub-pulmonic) dysfunction at time of surgery (Peng et al., 2014) suggesting these patients may be at risk for left (sub-pulmonic) ventricular failure and the need for possible ventricular mechanical support (Maly et al., 2015). The complexity of VAD support in these patients is further increased by the potential for cannula obstruction due to right ventricular trabeculations/moderator band (Agusala et al., 2010; Joyce et al., 2010) and the frequency of clinically significant tricuspid regurgitation in these patients (Graham et al., 2000; Peng et al., 2014).

The use of a TAH would alleviate the concerns mentioned above and also provide configuration flexibility, which is important to accommodate the variety of anatomic considerations in these patients. This was demonstrated by Morales et al. (2012) in a patient with ccTGA who presented with multi-system organ failure in the setting of severe biventricular dysfunction, severe aortic insufficiency and obstruction of a left-ventricle to pulmonary artery conduit. A TAH was implanted due to the multiple anatomic considerations noted above. He was discharged home within a month and was bridged to transplant during his sixth months of support. A recent report also described the use of a TAH in a patient with dextrocardia and ccTGA with ventricular septal defect, pulmonary stenosis and Ebsteinoid tricuspid valve who had recently undergone a Senning-Rastelli procedure due to pulmonary hypertension (Si et al., 2015). The patient had severe biventricular diastolic dysfunction and required extracorporeal membrane oxygenation (ECMO) support on post-operative day (POD) 1. The patient could not be weaned from ECMO and underwent TAH with Senning pathway reversal. He tolerated the procedure well and underwent heart transplant on POD day 8, followed by hospital discharge within a month.

## Failing fontan

Much has been written about the long term outcomes of the Fontan circulation including end-organ dysfunction, and its eventual morbidity and mortality. Generally, there is an obligate increase in central venous pressure with passive flow into the lungs and decreased ventricular preload. This is driven by the initial anatomic considerations as well as inefficient flow dynamics (Whitehead et al., 2007), atrial arhrythmias (Khairy et al., 2008; d'Udekem et al., 2014; Quinton et al., 2015), thromboembolism (Khairy et al., 2008), abnormal pulmonary vascular remodeling (Ridderbos et al., 2015) atrioventricular valve dysfunction, relaxation abnormalities and systolic dysfunction (Piran et al., 2002; Eicken et al., 2003). The culmination of these abnormalities leads to a significant burden of end organ dysfunction (Dimopoulos et al., 2008; Rychik et al., 2012; Schumacher et al., 2014) and mortality (Khairy et al., 2008).

The multiple levels of organ and cardiovascular dysfunction that contribute to Fontan failure (Frazier et al., 2005; Morales et al., 2011; d'Udekem et al., 2014) complicate the discussion regarding the choice and potential benefits of mechanical circulatory support in these patients. For example, while a systemic VAD will improve Fontan physiology when the primary abnormality is systemic ventricular dysfunction the situation is more complex when failure is multifactorial, which is more often the case (Khairy et al., 2008). Pulmonary vascular remodeling (Khambadkone et al., 2003; Ridderbos et al., 2015) and diastolic abnormalities (Anderson et al., 2008) often conspire to limit pulmonary blood flow and assessing the contributions of each is difficult at best. One process may also exacerbate the other as cavopulmonary associated power loss may lead to chronic underfilling (Haggerty et al., 2015) and worsening diastolic dysfunction. The degree of diastolic dysfunction may also be underappreciated by testing at rest as Fontan patients may have limited inotropic reserve (Senzaki et al., 2006). A recent report from the Pediatric Heart Network Investigators suggests that while desaturation and inotropic reserve may limit exercise function, impaired stroke volume reserve (Paridon et al., 2008) may be the primary driver of Fontan dysfunction as the inability to increase trans-pulmonary blood flow drives impaired systemic ventricular preload. This is in keeping with data suggesting power loss within the Fontan worsens with activity (Khiabani et al., 2015). Thus, augmenting systemic ventricular output with a VAD alone may not improve the overall physiology and may lead to further increases in central venous pressure as trans-pulmonary blood flow remains the limiting factor.

In addition, the failing Fontan circulation is often addressed as a single state to which different therapies are applied. However, this is a misconception as early Fontan failure is very different than late Fontan failure. Each of these stages of Fontan failure probably require different therapies and applying one therapy to all of the stages will result in inconsistent results. Early failure of the Fontan Circulation is commonly noted by the development of re-entrant tachycardia or mild clinical symptoms. For those patients with an atrial-pulmonary Fontan or anatomic obstruction, this may be successfully addressed with a Fontan conversion. Those patients who start to develop atrial fibrillation and early signs of renal or hepatic dysfunction are in moderate failure and can still be successfully treated with cardiac transplantation (Kanter et al., 2011). There is growing evidence that VAD support can be successful in a subset of these patients (Halaweish et al., 2015; Jabbar et al., 2015), however, it remains unclear when VAD implantation should be considered and what percentage of patients will benefit from systemic VAD alone. Patients with isolated, or predominant ventricular failure, are likely to benefit from VAD alone, however, Fontan failure is commonly multifactorial. A VAD will surely not help the clinical situation if the end-diastolic pressure of the systemic ventricle is not high (at least above 12 mmHG). Some centers have utilized a sub-pulmonary assist device either alone (Prêtre et al., 2008) or as part of a “biventricular” support strategy (Nathan et al., 2006; Valeske et al., 2014) in circumstances where the pulmonary vascular bed remained a limiting factor and CVP remained elevated, however, the results are mixed and the medium to long term outcome of this approach remains unclear. The addition of a sub-pulmonary assist device to force blood through an abnormal pulmonary vascular bed and into an often restricted systemic ventricle in the setting of heart failure is unlikely to be a successful long-term support strategy. On the other hand, the application of a sub-pulmonary support system very early in the clinical course, prior to the development of multi-organ dysfunction, may be able to avoid many of these concerns and is the topic of ongoing research (Di Molfetta et al., 2015). Those patients presenting with severe failure of their Fontan circulation, who have developed marked end-organ dysfunction, protein losing enteropathy, and/or plastic bronchitis are not good transplant candidates. It is these patients who may benefit from the TAH as it not only provides a supra-physiological cardiac index (often over 4 L/min/m^2^) but does so with a low CVP, something rarely seen with VADs or even early after transplantation. It is this immediate improvement in cardiac output in the environment of a decongested venous system that may allow recovery of renal (Ryan et al., 2015) and liver disease previously thought not to be reversible and thus improve transplant candidacy/mortality (as has been demonstrated in VAD use in adults with end-stage heart failure Russell et al., 2009).

The feasibility of using a TAH in the failing Fontan has been demonstrated in a recent case report by Rossano et al. (2014). The team placed a TAH in a 13 year old with pulmonary atresia with intact ventricular septum who presented *in extremis* with respiratory failure, hepatic dysfunction, and plastic bronchitis. A 70-cc TAH was placed after creating a “neo-right atrium.” The patient recovered end organ function and was able to ambulate prior to transplantation on POD 61. This case was also significant because it demonstrated the possibility of constructing a capacitance chamber in patients without two adequate AV valves. This is possible because the TAH does not generate significant “suction” which would collapse the capacitance chamber. That said, the mass of the device compressing the neo-atrium is a significant concern and may complicate this support strategy. It remains to be seen if a reproducible and efficient TAH implantation method can be developed that will function for the vast majority of Fontan anatomies.

## Virtual fit

The TAH presents a number of potential advantages to VAD support in patients with congenital abnormalities and with complex hemodynamics, anatomic abnormalities or previous palliations for the reasons mentioned above; however, the implantation is technically more challenging than implantation of a VAD. Assessing thoracic anatomy and device fit has been a concern since the early development of artificial hearts (Jacobs et al., 1978). This issue is particularly relevant in small adults (i.e., women) and adolescents. Historically, the 70 cc TAH was limited to adults or adolescents with a minimum 10 cm distance from the anterior surface of the T10 vertebral body to the sternum in order to ensure device alignment and to prevent kinking or obstruction of venous structures and the outflow grafts. Size based fit recommendations were subsequently developed (Copeland et al., 2004). Population based size and anatomic assumptions regarding chest shape/size, heart size and arterial relationship have given rise to patient specific virtual implantation based on cross sectional imaging (Chatel et al., 1993; Zhang et al., 1999; Dowling et al., 2004; Moore et al., 2014). Understanding patient specific anatomy and device fit is likely to be even more significant in patients with CHD given the range of chamber, valve and great vessel anomalies across the disease spectrum.

## Syncardia^®^ 50 cc clinical trial

**Figure 1:**
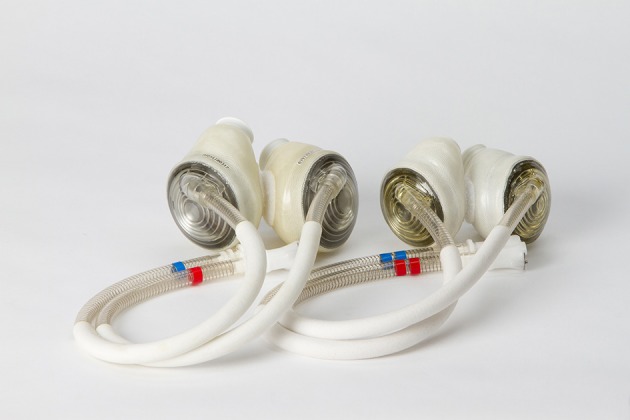
**Syncardia TAH 70 cc (left) and 50 cc (right) devices**.

While virtual fit may expand the number of patients who may benefit from a TAH, there was a clear need for a smaller device to accommodate small adults (especially women) and adolescents who are too small for the 70 cc TAH. Syncardia® has now developed a 50 cc pump which is 30% smaller by volume and is now enrolling children and adults in a bridge to transplant clinical study (Figure 1). The study will have both pediatric (age 10–18 years) and adult (19–75 years) arms. There are also secondary study arms for patients who would not qualify for study inclusion due to ECMO (either V-A or V-V) support >3 days or only 1 functioning AV valve (both of which are relevant to patients with CHD). Importantly, inclusion in the study is not based solely on patient BSA or thoracic measurement in a single plane. Patients can enroll in the study if virtual implantation suggests device implantation is feasible. Preoperative virtual implantation may alter device selection in up to ~33% of patients and may help to identify patients with a BSA <1.2 m^2^ who are candidates for implantation (Moore et al., 2016).

## Conclusions

The last few decades have seen a dramatic improvement in clinical outcomes among patients with CHD. These improvements are most pronounced in young children and those with severe CHD (Moons et al., 1970; Nieminen et al., 2007; Khairy et al., 2010) which were historically almost uniformly fatal. While early survival and improved quality of life are now the rule, many of the surgical interventions employed for repairing CHD are palliative rather than curative. This has shifted the burden of heart failure and cardiac mortality to the teenage and early adult years (Zomer et al., 2013; Diller et al., 2015). While the availability of VAD technology has improved overall heart failure outcomes, the proportion of patients with CHD who are supported by a VAD noticeably trails the proportion of patients with cardiomyopathy who are supported by a VAD (Davies et al., 2011). This is especially concerning given children (Almond et al., 2009; Jeewa et al., 2014; Zafar et al., 2015a) and adults (Davies et al., 2011) with CHD have higher waitlist mortality. The differences in waitlist mortality are multifactorial, but have been attributed to both limited access to VAD technology (Gelow et al., 2013) and sub-optimal outcomes once a VAD has been implanted in patients with CHD (Davies et al., 2011; Everitt et al., 2011). Recent use of the TAH (Morales et al., 2012; Rossano et al., 2014) has highlighted how the TAH may be able to overcome some of the historical limitations to VAD use in CHD, although long-term data is needed. The TAH has the ability to improve end-organ function by simultaneously increasing cardiac output and lowering CVP, to address restrictive heart failure, and to provide a reliable, pulsatile pump that can address the multiple levels of failure that lead to Fontan failure. The TAH pumps may also be placed in a variety of orientations (criss-cross, parallel, other) which allows surgical flexibility and innovation to account for the anatomic variation associated with CHD. Ultimately, these characteristics may make the TAH the most effective bridge to normal physiology for patients with complex CHD who have been only partially medically or surgically palliated since birth.

## Author contributions

CV provided conception/design of the review, contributed to critical drafting and revision of the manuscript and provided final approval for the manuscript. DM provided conception/design of the review, contributed to critical drafting and revision of the manuscript and provided final approval for the manuscript.

## Disclosure:

DM has served as a consultant for Syncardia. DM is also a consultant for HeartWare and Berlin Heart.

### Conflict of interest statement

The authors declare that the research was conducted in the absence of any commercial or financial relationships that could be construed as a potential conflict of interest.
